# Pacemaker Implants in Children and Adolescents with Chagas Disease in Brazil: 18-Year Incidence

**DOI:** 10.5935/abc.20170074

**Published:** 2017-06

**Authors:** Carolina Christianini Mizzaci, Thiago Gonçalves Schroder e Souza, Gabriel Pelegrineti Targueta, Ana Paula Frederico Tótora, Juan Carlos Pachón Mateos, José Carlos Pachon Mateos

**Affiliations:** Instituto Dante Pazzanese de Cardiologia, São Paulo, SP - Brazil

**Keywords:** Retrospective Studies, Pacemaker,Artificial, Child, Adolescents, Chagas Disease, Chagas Cardiomyopathy, Epidemiology

## Abstract

**Background:**

Chagas disease continues to be a serious public health problem, and accounts for 25-30% of the indications for cardiac stimulation in Brazil.

**Objective:**

To assess clinical and epidemiological characteristics of patients with Chagas disease, younger than 18 years, who had undergone pacemaker implantation in Brazil between 1994 and 2011, and its temporal trend.

**Methods:**

This was a cross-sectional analysis of data from the Brazilian Pacemaker Registry database. The following variables were analyzed: year when pacemaker was implanted, location, age, sex, ethnic group, functional class and the main electrocardiographic findings at baseline.

**Results:**

In a total of 183,123 implants performed between 1994 and 2011, 214 implants of cardiac stimulation device in Chagas disease patients aged younger than 18 years were identified. Mean age at implantation was 5.6 ± 6.2 years. Second- and third-degree atrioventricular blocks corresponded to 71% of indications for pacemaker implantation. Fifty-six percent of the procedures were performed in the southeast region. Regarding the total number of pacemaker implants per year, there was a remarkable increase in the implants for all causes. However, time series analysis of the implants in Chagas disease patients younger than 18 years revealed a significant reduction in the annual number of implants.

**Conclusion:**

There has been an important reduction in the number of pacemaker implantations among children and adolescents with Chagas disease, suggesting a reduction in the vertical transmission of the parasite.

## Introduction

Endemic in South America and emerging in Europe and in the United States, Chagas disease continues to be a serious public health problem. Estimates indicate that there are 2.9 - 7.2 million people with Chagas disease in Brazil,^1^ which accounts for approximately 6 thousand deaths per year.^2,3^ According to the Brazilian Pacemaker Registry (BPR), 25%-30% of cardiac stimulation are performed for Chagas disease in Brazil.^4^

In addition to transmission via infected feces of the hematophagous triatomine insect, Trypanosoma cruzi may also be transmitted by blood transfusion, consumption of contaminated food or drinks, and congenital transmission (from mother to child).^5^ Due to a more effective control of both vector and transfusional transmission, congenital route has emerged as the most important way of transmission in most endemic areas.^6,7^

Prevalence of *T.cruzi* infection in pregnancy varies from 1% to 40%,^8-12^ and congenital transmission may reach 28.6%.^7^ Recent estimates indicate that annually, more than 14 thousand babies are born with congenital Chagas disease in Latin America. A Brazilian study conducted between 2001 and 2008 on 105 thousand children aged from 0 to 5 years living in rural areas reported a 0.03% prevalence of *T. cruzi,* 0.02% for probable congenital transmission and 0.01% for vectorial transmission.^13^

Although most cases of congenital infection of *T.cruzi* are asymptomatic, it may cause premature death, low birth weight, stillbirths and clinical manifestations of Chagas disease at birth.^14,15^ Since congenital transmission cannot be prevented, early diagnosis and treatment of congenital cases are the main goals of the programs for Chagas disease control.^16,17^

Considering changes in demography and transmission pathways, in particular the rising importance of vertical transmission, information on how these changes may affect patients’ treatment and outcome are still scarce. Therefore, aiming to contribute to the knowledge on the theme, the objective of this study was to evaluate clinical and epidemiological characteristics of Chagas disease patients younger than 18 years, who had undergone a permanent pacemaker implantation in Brazil in the period between 1994 and 2011.

## Methods

Data of the BPR database were analyzed in this study. This database system, officially created by the Ministry of Health decree no. 41, of December 17^th^, 1994, is maintained by the Department of Artificial Cardiac Stimulation of the Brazilian Society of Cardiology Surgery. The system holds information of permanent cardiac stimulation procedures performed in Brazil by means of a standardized, specific form about generator implants performed in the country. Completed forms were forwarded to the central, where the information was registered.

The following variables were analyzed: year when implant was performed, place of origin, age, sex, ethnic group, heart failure functional class according to the New York Heart Association (NYHA) criteria, and the main electrocardiographic finding that indicated the need for a pacemaker.

Categorical variables were expressed as absolute and relative frequencies, and continuous variables as mean and standard deviation. Statistical analysis was performed using the SPSS (*Statistical Package for the Social Sciences*) software.

Temporal variation in the number of pacemaker implants was assessed by the Jonckheere’s trend test, and the alpha error was set at 0.05.

## Results

Between 1994 and 2011, a total of 183,123 patients undergoing first pacemaker implantation were identified. Of this total, 35,204 were performed in patients with Chagas disease, and 214 of them consisted of surgical implantation of cardiac stimulation devices in patients aged 17 years or less.

**Figure 1:**
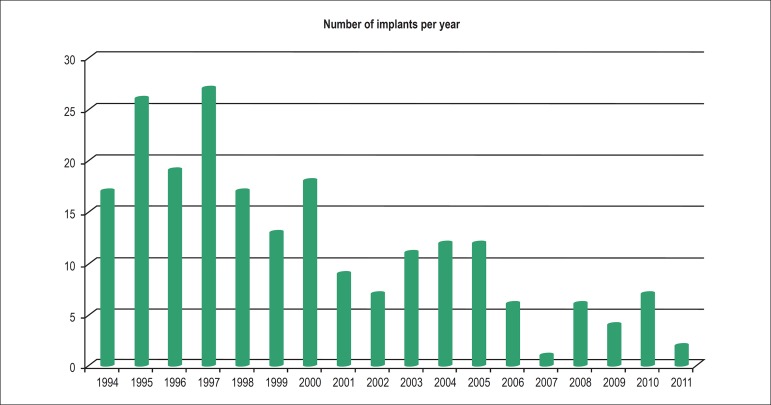
Annual distribution of pacemaker implantations in Chagas disease patients younger than 18 years.

Source: Brazilian Pacemaker Registry.

In the group of patients with Chagas disease younger than 18 years, who had undergone a pacemaker implant, mean age at procedure was 5.6 ± 6.2 years. Forty-five percent of these patients were women (5.2 ± 5.8 years), and 55% were men (6.1 ± 6.5 years). The Figure 1 shows the absolute frequency of implants performed per year throughout the period assessed (18 years), with a remarkable reduction in the number of implants. Mean number of implants was 20.6 implants/year in the first triennium (1994-1996), and 4.3 implants/year in the last triennium, indicating a 79.1% decrease between these periods.

Distribution of procedures by geographic area revealed a considerable diversity. Most patients came from the southeast of Brazil; in fact, most of Chagas disease patients were from this region (55.6% of the cases), followed by the central west region (25.7% of the cases).

Regarding ethnic characteristics of the patients, most of them were white (49.5% of the implants), followed by mestizos (21.1%) and black individuals (14%). With respect to symptoms, most patients were NYHA class III and IV. One hundred patients (46.7%) were symptomatic during moderate/little efforts and 68 (31.8%) had symptoms at rest (Table 1).

**Table 1:** Baseline characteristics of Chagas disease patients younger than 18 years who had undergone implantation of cardiac stimulation devices between 1994 and 2011

Patients (n)		214
Age (years)		5.62 ± 6.2
Sex	Male	118 (55.2%)
	Female	96 (44.8%)
Federative unit of origin	Sao Paulo	59 (27.6%)
	Minas Gerais	59 (27.6%)
	Goias	36 (16.8%)
	Distrito Federal	19 (8.9%)
	Parana	13 (6.1%)
	Bahia	8 (3.7%)
	Alagoas	5 (2.3%)
	Pernambuco	5 (2.3%)
	Others	10 (4.7%)
Ethnic group	White	106 (49.5%)
	Mestizo	43 (20.1%)
	Black	30 (14.0%)
	Not declared	35 (16.3%)
Symptoms	Asymptomatic	19 (8.9%)
	Symptoms during great efforts	22 (10.3%)
	Symptoms during light/moderate efforts	100 (46.7%)
	Symptoms during rest	68 (31.8%)
	Not declared	5 (2.3%)

**Figure 2:**
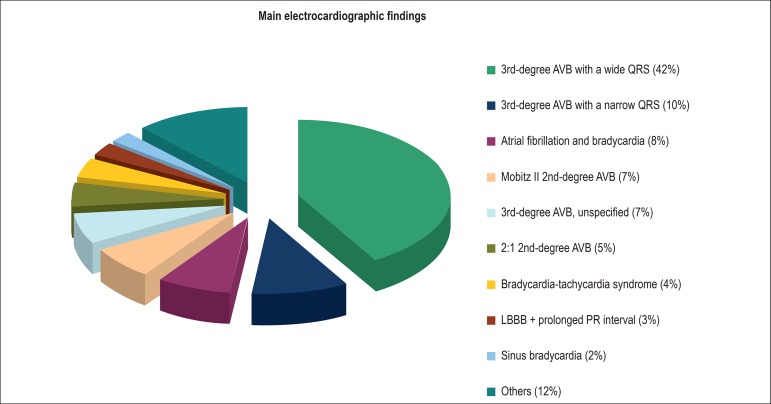
Electrocardiographic fndings suggesting the need for pacemaker implantation in Chagas disease patients younger than 18 years. AVB: atrioventricular block; LBBB: left bundle branch block.

Source: Brazilian Pacemaker Registry.

Seventy-one percent of the electrocardiographic indications for implantation of cardiac stimulation system were second- and third-degree atrioventricular block (AVB). Most of them were complete AVB with a wide QRS complex (42% of incidence), whereas complete AVB with a narrow QRS complex was reported in 10% of patients (Figure 2).

**Figure 3:**
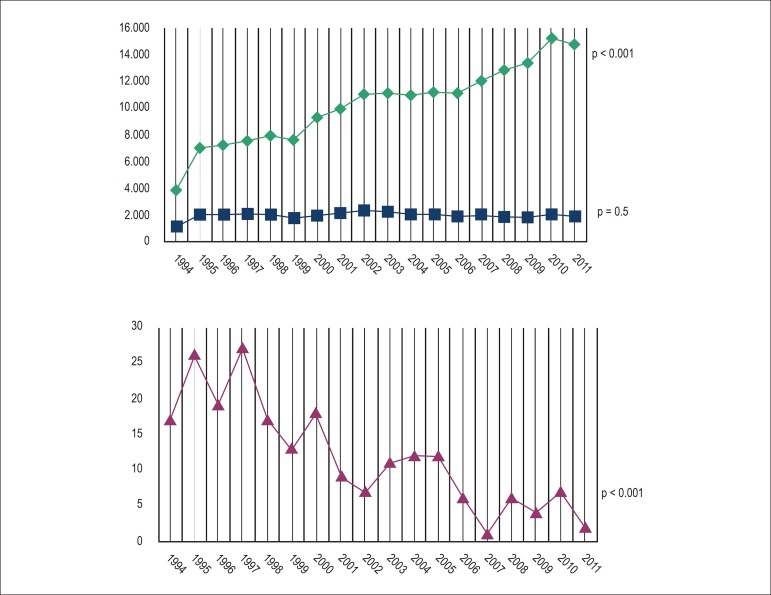
Comparative graph of total pacemaker implants for all causes performed in Brazil per year (diamonds), total number of pacemaker implants in Chagas disease patients (square) and total pacemaker implants in Chagas disease patients younger than 18 years (triangle). P value of variation trend in each series through the years.

Source: Brazilian Pacemaker Registry.

Considering the total number of pacemaker implants per year (Figure 3), there was a relevant, statistically significant increase in the number of implants for all causes. Time series analysis of the number of implants in Chagas disease patients of all ages showed a slight, non-significant variation. The possibility that this variation has occurred by chance cannot be ruled out (p trend = 0.5). Nevertheless, time series analysis of the implants in Chagas disease patients younger than 18 years revealed a significant reduction in the number of implants through the years (p trend < 0.001) (Figures 1 and 3).

## Discussion

In 1999, the Pan-American Health Organization (PAHO) declared that Triatoma infestans, the vector insect of *T. cruzi*, had been completely eliminated from human dwellings in Brazil, Chile, Uruguay, and large portions of Argentina, Bolivia, and Paraguay.^19^ Nevertheless, despite recent advances in the control of T.cruzi transmission, Chagas disease continues to be an important public health problem in Latin America, with an annual impact of 430,000 DALYS (Disability-Adjusted Life Years) in the region.^18^ Two hypotheses may be raised from this fact:

1. there have been continuous or increasing expenses on the treatment of chronic Chagas disease, particularly on patients with chronic Chagas cardiomyopathy (CCC), or
2. the parasite transmission modes have not been effectively controlled yet, which causes concern regarding blood transfusion transmission and vertical transmission of *T.cruzi*.

Data of the Brazilian Pacemaker Registry reflect CCC morbidity and hence yield useful information. Approximately 20% of infected patients develop CCC, and are at high risk for AVB and cardiac sudden death.^20^ Interestingly, here we describe that, despite the increase in the number of pacemaker implants in Brazil, the number of procedures performed in Chagas disease patients per year did not change in the same period. This reflects a relative reduction of CCC and increase of other causes - such as senile degeneration of the conduction system - as indications for artificial stimulation of the heart. This finding may be due to a more effective control of vectorial and transfusional transmission of Chagas disease, as well as to an increase in life expectancy in the Brazilian population.^21^

Our most important finding was the drastic decrease in the use of artificial cardiac stimulation in individuals younger than 18 years, which may suggest a better control of Chagas disease transmission in Brazil in the last decades. As previously mentioned, this result may be partly explained by the control of the vector. However, the decrease in blood transfusion transmission in addition to the continuous, effective control of vertical transmission of *T.cruzi* may have also contributed to it. At the end of the eighties, screening of blood donors for *T.cruzi* infection became compulsory in Brazil and, before this measure was implemented, approximately 20,000 new cases of Chagas disease were attributable to transfusional transmission per year. Today, the estimated risk of contamination of blood components by *T.cruzi* may be lower than 1 in 1,000,000 of transfusions.^21^

Although the relevance of vertical transmission of Chagas disease has increased since the control of other transmission modes of the disease in Brazil, there are no conclusive data about its real magnitude. According to a recent systematic review,^1^ the infection prevalence among pregnant women varies from 0.1 to 8.5%, and the vertical transmission rate varies from 0 to 5.2%. The decrease in vertical transmission is also corroborated by the fact that conduction system diseases require years for its establishment, occurring in last stages of CCC.

Another finding that deserves attention is the uneven geographical distribution of the number of pacemaker implants across the national territory, not following the regions of higher prevalence of CCC. In addition to the concentration of main public health services in the big cities, the lack of trained experts in artificial cardiac pacing in children could also lead to the concentration of these procedures in tertiary health centers in capitals like Sao Paulo.

This study has some limitations inherent to the study design. First, accuracy of data may be affected by the inter-subject variability of individuals responsible for feeding the database. Second, the study only allows us to formulate causal hypothesis related to the management of Chagas disease in the last years, not only for the retrospective nature of the study, but also for the adoption of a variable that does not represent the whole. Despite these considerations, we believe that our study provide useful information for the planning of health systems.

## Conclusion

There has been an important reduction in the number of pacemaker implantations among children and adolescents in Brazil, suggesting a better control of Chagas disease transmission in Brazil in the last two decades and a reduction in the vertical transmission of the parasite.
